# Abdominal cystic lymphangioma in a term newborn: A case report and update of new treatments

**DOI:** 10.1097/MD.0000000000005984

**Published:** 2017-02-24

**Authors:** Ilaria Amodeo, Giacomo Cavallaro, Genny Raffaeli, Lorenzo Colombo, Monica Fumagalli, Riccardo Cavalli, Ernesto Leva, Fabio Mosca

**Affiliations:** a Neonatal Intensive Care Unit; b Pediatric Dermatology Department; c Department of Pediatric Surgery, Fondazione IRCCS Cà Granda Ospedale Maggiore Policlinico, Università degli Studi di Milano, Milan, Italy.

**Keywords:** cystic lymphangioma, lymphatic abnormalities, sclerotherapy, sirolimus

## Abstract

**Introduction:**

Lymphatic malformations are benign anomalies derived from the abnormal development of lymphatic channels. Usually asymptomatic, they can cause compression on adjacent structures or present acute complications (bleeding or infection). Small asymptomatic lesions can be conservatively managed since the possibility of spontaneous regressions is described, while symptomatic lesions require active management. Less invasive therapeutic options are now preferred instead of surgery (sclerotherapy, laser therapy). However, there are not uniform therapeutic protocols.

**Case Report:**

We present the case of a term newborn with an abdominal cystic lymphangioma extending from the umbilical to the right inguinal area, reaching the medial surface of the right tight. Despite its large dimensions, which classically request surgical management, the patient was by chance asymptomatic, and the mass did not determine compression on the surrounding organs. Therefore, conservative management was tried, and a close clinical and radiological follow-up was started. This approach permitted a spontaneous regression of the mass and to avoid major surgical intervention.

**Conclusion:**

Our purpose is to underline the possibility of conservative management of the major multicystic masses and to focus on less invasive therapeutic options, like sclerotherapy, oral therapy, and laser therapy.

## 1 Introduction

Vascular malformations are described as a group of anomalies due to a disembryogenetic process, which can result in arterial, venous, capillary, lymphatic, or combined malformations.^[1]^

Lymphatic malformations (LMs) are considered to be rare and benign anomalies caused by the defective embryological development of the primordial lymphatic structures. They consist of dilated lymphatic channels forming multiple cysts. LMs can be classified into macrocystic, microcystic, or mixed cystic lesions.^[1]^

Macroscopically, they can give rise to the formation of masses of different sizes that depending on their location can be variably appreciated through deep palpation as soft, non-pulsatile, and painless masses.^[2]^ The overlying skin is normal, but it can also have a bluish hue or contain pink vesicles, that can appear similar to capillary malformations, angiokeratomas, or angiomas. Cutaneous manifestations are often the main clue that reveals the presence of further complex underlying condition, with deeper lesions involving different tissue planes.^[3]^

The cervicofacial region is most affected (48%). LMs can also be found in the trunk, axilla, and extremities (42%). The mediastinal and abdominal locations are rare (10%). Half of these lesions are typically present at birth and are often diagnosed prenatally. Nevertheless, 90% are diagnosed at 2 years old. Only a minority of cases cannot be detected due to their deep localization or absence of symptoms.^[2]^

Here, we discuss the case of a term newborn with an abdominal cystic lymphangioma, diagnosed through prenatal ultrasound screening, and its management.

## 2 Case report

### 2.1 Presenting concerns

A female caucasian baby was born by cesarean section at 39 weeks of gestation to a 37-year-old mother. Family medical history was unremarkable. The pregnancy had a normal course until 32 weeks of gestation when an obstetric ultrasound (US) showed the presence of a cystic abdominal mass of about 3 cm in diameter, localized between the bladder and the umbilicus, which was suggestive of an urachal cyst without other structural alterations of the urinary apparatus. At birth, APGAR score was 9/10 and birth weight 3200 g.

### 2.2 Clinical findings

At physical examination, a vascular lesion (4 cm × 4 cm) was detected in the right lower abdominal quadrant. The presence of a soft suprapubic mass was also appreciated, without alterations of the overlying skin. The patient was otherwise asymptomatic.

The dermatologist confirmed the diagnosis of complex vascular malformation: it consisted in a venocapillary superficial lesion associated with multicystic lymphangiomatous lesions in the subcutaneous tissues, involving the right lower abdomen and the homolateral inguinal area and tight. Also, a small angiomatous lesion of the right first toe with no dysmetria of the affected limb was detected at physical examination.

### 2.3 Timeline

Table 1 summarizes the main clinical and radiologic findings in our patients, which led to the diagnosis of multicystic lymphangioma, and the major steps in the management and follow-up.

**Table 1:**
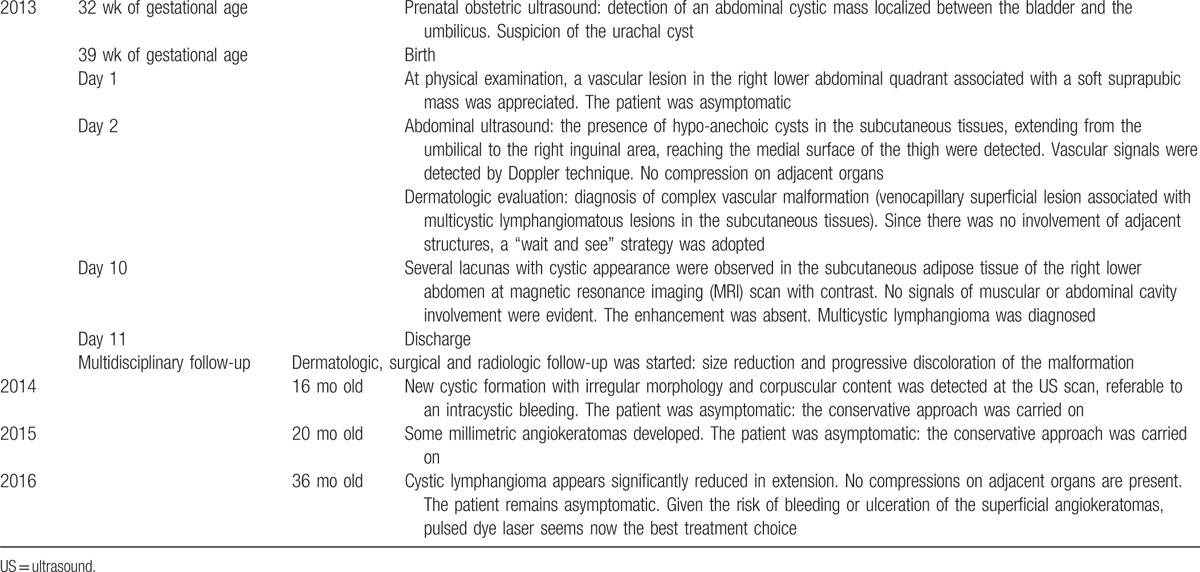
Timeline.

### 2.4 Diagnostic focus and assessment

**Figure 1:**
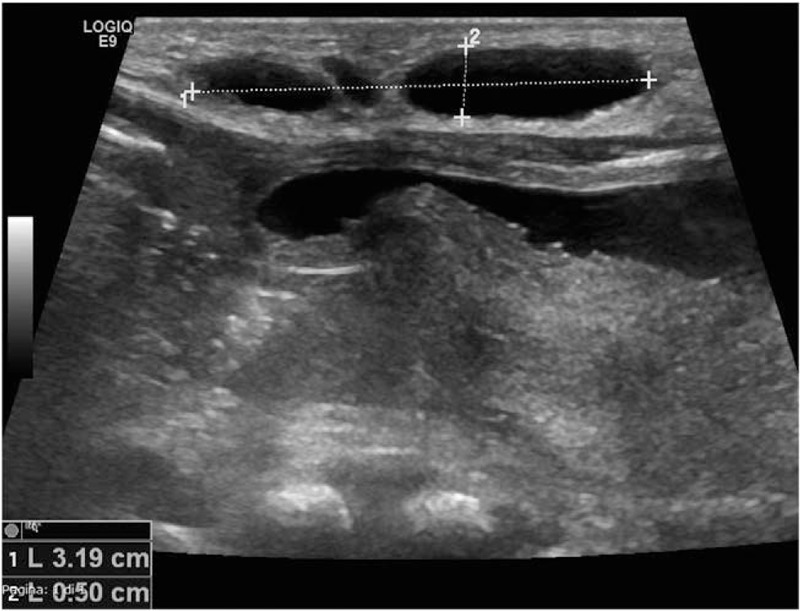
Abdominal ultrasound scan showing subcutaneous cysts. 1: maximum diameter 3.1 cm, 2: minimum diameter 0.5 cm.

**Figure 2:**
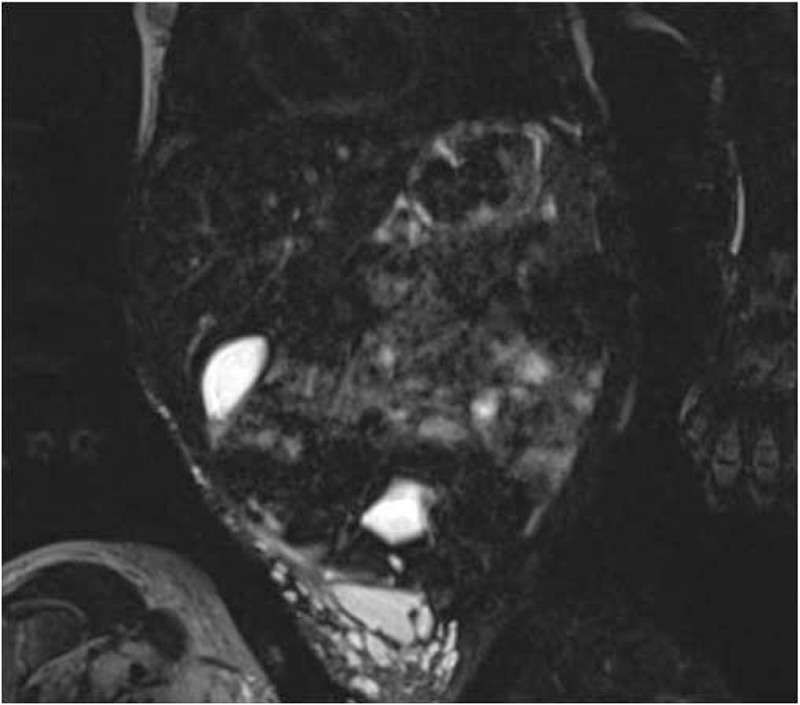
MRI scan with contrast in the coronal plane. Cysts can be observed in the subcutaneous adipose tissue of the right lower abdomen and right thigh. MRI = magnetic resonance imaging.

On the second day of life the abdominal US was performed, and no congenital urachus abnormalities were detected, but rather some hypo-anechoic cysts of about 3.1 × 0.5 cm that extended from the umbilical to the right inguinal area, reaching the medial surface of the thigh, with a little extremity just above the homolateral knee (Fig. 1). The Doppler technique showed some vascular signals from the adipose tissue where these cystic lesions were located. A magnetic resonance imaging (MRI) scan with contrast was then performed. In consideration of the age of the patient, sedation was needed during the exam. At MRI, several lacunas with cystic appearance were observed in the subcutaneous adipose tissue of the right lower abdomen. These structures had variable size (maximum 3 cm of diameter) and were hypointense in T1 and hyperintense in T2 sequences. Cysts seemed to be restricted to the subcutaneous tissues since no signals of muscular or abdominal cavity involvement were evident. After the intravenous contrast administration, no significant enhancement of the cysts’ content was seen. All MRI characteristics confirmed the diagnosis of multicystic lymphangioma (Fig. 2).

### 2.5 Therapeutic focus and assessment

As surrounding abdominal organs were not involved and compressive complications on adjacent organs were not present, given the possibility of spontaneous regression of the lesion, a “wait and see” strategy was adopted, and the patient was closely monitored through a multidisciplinary follow-up program.

### 2.6 Follow-up and outcomes

A size reduction and progressive discoloration were macroscopically appreciated, and progressive regression was confirmed through serial US evaluations.

Sixteen months later, the presence of a new cystic formation (3.6 cm × 2.1 cm × 1.9 cm) with irregular morphology and corpuscular content was detected at the US scan, referable to an intracystic bleeding. Since the patient was asymptomatic and no other complications were detected, the conservative approach was carried on.

During the following months, some millimetric angiokeratomas developed.

Almost 3 years later, the cystic lymphangioma appears significantly reduced in extension, and the cysts now have a maximum diameter of 0.8 to 1 cm. No alterations or compressions of adjacent organs were present. The patient is 3 years old, has normal neurodevelopment and does not have any abdominal disturbances. No compression on adjacent organs nor dysmetria at the inferior limbs is present.

However, given the risk of bleeding or ulceration of the superficial angiokeratomas, pulsed dye laser seems now the best treatment choice.^[4,5]^

## 3 Discussion

Intra-abdominal cystic lymphangiomas are a rare type of lymphangioma. Their clinical manifestations are highly polymorphic. A large tumor volume usually appears as a palpable mass which can cause abdominal pain (in older patients), an increase in waist circumference and, in severe cases, acute abdominal emergencies like intestinal obstruction or volvulus. The two most common acute complications are bleeding and infection. Bleeding is typically intra-cystic with a consequent bluish discoloration of the LM, swelling, and pain. Infection can progress rapidly to sepsis and hospitalization is often necessary to provide an adequate intravenous antimicrobial therapy.^[6]^

As associated manifestation, superficial angiokeratomas can develop which can easily bleed or ulcerate under mechanical stimuli.^[4]^

Ultrasound examination is an excellent modality to detect these lesions, and it is the gold standard to monitor their clinical evolution. The computed tomography (CT) and MRI scanner are a valuable initial diagnostic tool. However, MRI is the most informative exam. Histologic confirmation of LMs is rarely necessary.

The goal of LM management and treatment is to maintain the functionality, to preserve the aesthetic integrity, and to control associated symptoms. Given the large heterogeneity of LM, therapy should always be individualized.

Surgical excision has been historically considered the treatment of choice for macrocystic/unicameral LMs.^[7]^ It consists in the complete resection of the lesion but presents a high complication rate (in particular bleeding, iatrogenic damages, and deformity) without guaranteed success. In multicystic/combined LMs, resection is usually incomplete since lesions involve several tissue planes, with a recurrence rate of 35% to 64%, which decreases to 17% to 22% in case of total excision.^[8]^ For these reasons, surgical excision is reserved for selected cases such as well-localized and unicameral LMs for which it may result to be curative, obstructing masses, which compromise vital functions, complicated or symptomatic macrocystic/combined LMs unsuitable for sclerotherapy.^[6]^

Today, less invasive therapeutic options are preferred (Table 2). Small asymptomatic lesions, with a variable frequency up to 45%, spontaneously regress and can be carefully monitored. Therefore, surgery is usually postponed as much as possible, at least until the age of 18 to 24 months.^[6,9–11]^

**Table 2:**
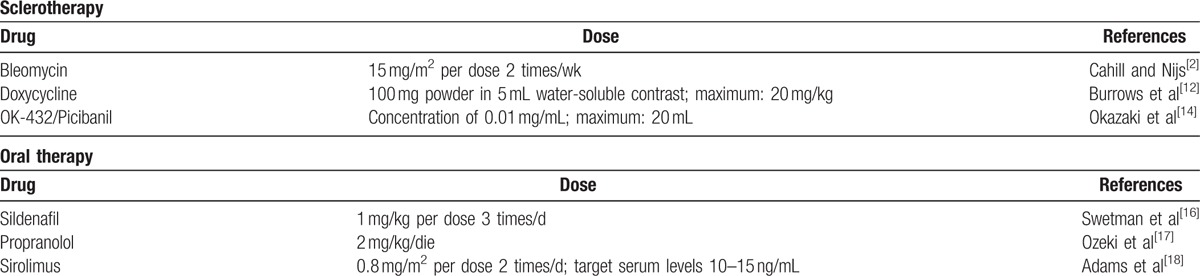
Therapeutic scheme of the principal pharmacological agents used for the treatment of lymphatic malformations.

Sclerotherapy has become the main therapeutic choice. It consists of US-guided percutaneous cysts aspiration and subsequent injection of a proinflammatory substance (such as doxycycline, bleomycin, OK-432/Picibanil), which cause scarring of the cyst walls (for the therapeutic scheme, see the Table 2). Sclerotherapy has a high success rate with lower recurrence and complication rates, especially for microcystic LMs. The most common complication of sclerotherapy is skin ulceration (<5%).^[12]^

Doxycycline is the most common sclerosant agent used to treat LMs, with 70% to 100% response rate and 10% to 14% complication rate. Pain is the main side effect. Other systemic complications are hemolytic anemia, metabolic acidosis, hypoglycemia, and delayed neural complications.^[12]^

There have been reported deaths related to bleomycin sclerotherapy, and because of its known risk of pulmonary fibrosis, it is a less attractive sclerosing agent, although very effective.^[2,13]^

OK-432 has >80% success rate but causes systemic reactions such as fever, malaise, anorexia and cannot be administered in children with penicillin allergy.^[2,14]^

Laser treatment with the carbon dioxide or the Nd:YAG is reserved for associated superficial cutaneous/mucosal vesicles, especially in the management of acute bleeding in cutaneous or subcutaneous lesions.^[15]^ For superficial lymphatic malformations at high risk of bleeding, such as angiokeratomas, pulsed dye laser might be considered as a treatment option.^[4,5]^

New therapies continue to emerge for the treatment of LMs. Sildenafil, sirolimus, and propranolol are three oral medications that have been reported to be effective in the recent literature.

Case reports show that sildenafil can decrease LM size and alleviate associated symptoms.^[16]^ Propranolol is routinely used for infantile hemangiomas, recent studies have shown its efficacy in the treatment of some LMs, although some patients do not respond to treatment.^[17]^ Sirolimus is an efficacious and safe treatment for complex vascular anomalies, even when refractory to other treatment. Sirolimus is an mTOR inhibitor and exerts antiangiogenic activity in tumors by impairing vascular endothelial growth factor (VEGF) production. It has been successfully used to treat vascular anomalies in children and newborn. Described side effects are mucositis, hypercholesterolemia, headache, elevation of transaminases, neutropenia.^[18]^

New surgical techniques have also been utilized in the treatment of LMs. Ultrasound-guided liposuction was shown to be efficient and has been used in conjunction with sclerotherapy to treat multicystic LMs better.^[19]^

In this clinical case, the patient had a voluminous multicystic malformation involving the abdominal subcutaneous tissues from the mesogastric region to the right inguinal area, reaching the medial surface of the homolateral thigh. In such extended and multicystic lesions, surgical excision is frequently incomplete, and this implicates high risk of failure and high recurrence rate. Moreover, special considerations have to be taken into account for the neonatal patient, for example, the risks related to total anesthesia and the need for mechanical ventilation, the risk of major blood loss, infections, and iatrogenic injury. Pain control is also challenging. The postoperative scar and deformity after removal of the LM should be weighted. For all these reasons, we would have considered surgery only in case of compressive, symptomatic or complicated masses, trying to postpone the intervention as much as possible.^[6,8]^

In our patient, the lesions did not involve primary structures and did not cause compressive complications on adjacent organs. This favorable situation permitted us to choose a conservative management (“watchful waiting”), hoping in the spontaneous regression of the cysts, as suggested by the recent literature.^[11,20]^

Since it was a complex malformation, a close clinical and radiological follow-up was needed. As we expected, serial ultrasounds showed a progressive reduction of the cysts. Bleeding occurred just once, with no other complications associated. The patient remained asymptomatic for almost 2 years, and then some millimetric angiokeratomas developed. Angiokeratomas are superficial vascular anomalies with a tendency to bleeding and ulceration as a consequence of mechanical or traumatic stimuli. At 3 years of age, the patients started to attend the nursery school, and some minimal bleeding occurred. To avoid major complications, we decided to consider a non-invasive treatment. Although CO_2_ laser can achieve successful results, it has some disadvantages, such as the requirement of anesthesia and the risk of scarring.^[21]^ As reported in previous cases, pulsed dye laser is used to treat vascular lesions and lymphangioma circumscriptum with a lower risk of complication such as pigmentation or scarring.^[4,5,22]^ For these reasons, we opted for pulsed dye laser to treat the cutaneous manifestations and prevent local complications.

This clinical case demonstrates that, if the mass does not compromise vital function or undergoes acute complications, a conservative approach is a reasonable choice in the management of large and complex LMs. This approach permits to take advantage of spontaneous regression in most cases and to consider less invasive therapeutic options later.
